# Cell Group Recognition Method Based on Adaptive Mutation PSO-SVM

**DOI:** 10.3390/cells7090135

**Published:** 2018-09-12

**Authors:** Yue Wang, Xiaochen Meng, Lianqing Zhu

**Affiliations:** Beijing Key Laboratory for Optoelectronic Measurement Technology, Beijing Information Science and Technology University, Beijing 100192, China; wangyue_1231@sina.com (Y.W.); zhulianqing@sina.com (L.Z.)

**Keywords:** biomedicine, flow cytometry, fluorescent reagent, cell clustering, supervised clustering, adaptive mutation PSO-SVM

## Abstract

The increased volume and complexity of flow cytometry (FCM) data resulting from the increased throughput greatly boosts the demand for reliable statistical methods for the analysis of multidimensional data. The Support Vector Machines (SVM) model can be used for classification recognition. However, the selection of penalty factor *c* and kernel parameter *g* in the model has a great influence on the correctness of clustering. To solve the problem of parameter optimization of the SVM model, a support vector machine algorithm of particle swarm optimization (PSO-SVM) based on adaptive mutation is proposed. Firstly, a large number of FCM data were used to carry out the experiment, and the kernel function adapted to the sample data was selected. Then the PSO algorithm of adaptive mutation was used to optimize the parameters of the SVM classifier. Finally, the cell clustering results were obtained. The method greatly improves the clustering correctness of traditional SVM. That also overcomes the shortcomings of PSO algorithm, which is easy to fall into local optimum in the iterative optimization process and has poor convergence effect in dealing with a large number of data. Compared with the traditional SVM algorithm, the experimental results show that, the correctness of the method is improved by 19.38%. Compared with the cross-validation algorithm and the PSO algorithm, the adaptive mutation PSO algorithm can also improve the correctness of FCM data clustering. The correctness of the algorithm can reach 99.79% and the time complexity is relatively lower. At the same time, the method does not need manual intervention, which promotes the research of cell group identification in biomedical detection technology.

## 1. Introduction

Flow cytometry (FCM) is a precise instrument for quantitative analysis and sorting of suspended cells and particles. It plays an important role in genetics, oncology, immune cell subgroup analysis and so on [1]. Flow cytometry consists of four parts: flow chamber and liquid flow drive system, light source and optical system, signal collection and signal conversion system, and computer and analysis system. The test samples are usually cells stained with a variety of fluorescent dyes, such as single laser five colors, double laser four colors, three laser eight colors, etc., the latest is Beckman Kurt’s upgraded of four laser tricolor CytoFLEXS flow cytometry, the wavelength of the laser is 488 nm, 638 nm, 405 nm and 561 nm respectively [2], which greatly increases the dimension of the fluorescence signal parameter in the process of signal analysis. The method of manual gating with professional software is used intraditional analysis of flow cell data, which has complex operation, poor repeatability and high operating threshold. The analysis of multi-parameter flow data is limited. How to realize automation of data analysis has become a hot research topic for many experts and scholars.

With the rapid development of science and technology, machine learning algorithms have been continuously innovated and optimized, and have been widely used in many fields, such as image processing, big data mining, natural language processing, unmanned technology, artificial intelligence and so on [3,4,5]. The combination of machine learning algorithm and biomedical engineering technology will be a hot spot in the field of medicine and the development trend in the future. In recent years, some classification algorithms of machine learning have been used in biological cell recognition. For example, a static cytometry with wide-angle two-dimensional light scattering was developed by Xie, L.Y. et al. Using gray difference statistics (GLDS) algorithm combined with support vector machine (SVM) algorithm, the two-dimensional light scattering images are analyzed to realize the automatic classification of acute and chronic myeloid leukemia cells [6]; to recognize gene subset, particle swarm optimization algorithm and KNN were used by Kar, S. et al. The subclass expression of tumor subgroup was screened from microarray gene expression data, and the cancer type was diagnosed accurately by using related genes [7]; to identify the cancer cells, the system was constructed with support vector machine, random forest tree and Bayesian classifier by Hasan, M.R. et al. The performance of the system was compared, which laid the foundation for automatic screening and classification of tumors [8]; recognition and differentiation of cell image based on convolution Neural Network algorithm was introduced by Tao Yuan and Wang Jiafai [9]; a new algorithm based on error back propagation neural network (BPNN) and MSD analysis to trained the neural network was proposed by Dosset, P. and Rassam, P. et al., and they used cross-validated with SHO to realize automatic detection of biofilm diffusion mode [10]; a Classification method for the diagnosis of sperm Morphology by using Principal component Analysis and K-nearest neighbor algorithm was proposed by Li, J. et al. [11].

In this paper, a support vector machine algorithm of particle swarm optimization (PSO-SVM) based on adaptive mutation is proposed to solve the problem of random setting of SVM model parameters. The kernel function is selected to construct the SVM classifier, and then the SVM is optimized by using the adaptive mutation PSO algorithm to optimize the performance of the classifier. The classification correctness of SVM module is greatly improved.

## 2. Theory and Method

### 2.1. The Generation of FC Data

**Figure 1:**
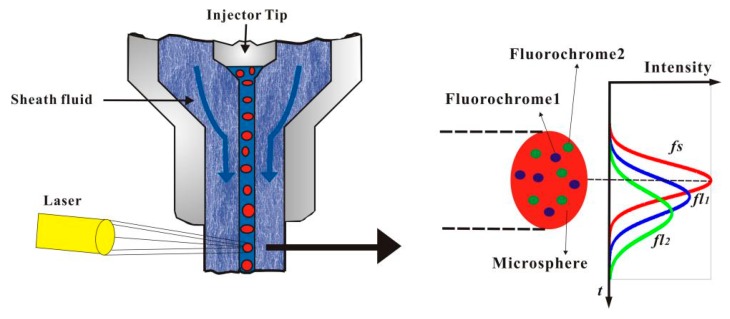
Generation principle of flow pulse signal.

The flow chamber part of flow cytometry is shown in Figure 1. A stable single cell layer is formed by the intersection of the sample fluid (or cell suspension) and the sheath fluid in the flow chamber. When irradiated with a specific wavelength laser, the forward scattered light *fs* and fluorescence signal *fl* will be produced. According to Mie scattering principle, the light intensity of the excited *fs* and *fl* has Gaussian-like trace property [12,13].

When a single cell is stained with two fluorescent reagents at the same time, different substances in the cell bind to different fluorescent reagents specifically. When the cell is irradiated by a laser of specific wavelength, two fluorescent signals *fl*_1_ and *fl*_2_, will be produced. Each pulse signal contains three parameters: the height, width and area, which of forward scattering light signal and fluorescence pulse signal are the basic composition of flow data. The detector converts *fs* into electrical signals. The *fl* is collected by the concentrator, and the *fl* of different colors is turned to different photomultiplier detectors by a two-color reflector, which also converts the *fl* signal into an electric signal. These electrical signals are then digitalized and entered into the computer and stored for cell analysis or sorting. Cells are stained with a variety of fluorescent reagents. The more kinds of fluorescent reagents, the more characteristic parameters of cells. In other words, the higher the dimension of flow data, the greater the difficulty of processing.

### 2.2. The Principle of Support Vector Machine Algorithm

**Figure 2:**
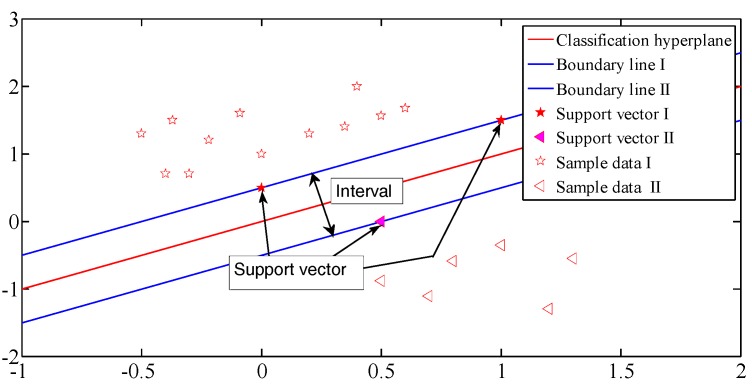
Support vector machine classification schematic diagram.

SVM algorithm is first used to solve the two-classification problem. When the sample data is linear, the SVM algorithm is the best hyperplane process to divide the two kinds of data in the plane space. For example, when the sample is linearly separable, SVM classification principle is shown in Figure 2. Two blue lines are at the edge of two types of data, the point on the edge line is called support vector, and the red line is the optimal classification line. It is equal to the distance between two edge lines, so the constraint of determining the optimal classification line can be transformed into maximizing the classification interval.

The optimal classification line:

(1) $$w^{T}x+b=0$$

The lines in which the support vector is located are:

(2) $$\begin{array}{c} w^{T}x+b=1\\ w^{T}x+b=-1 \end{array}$$

The classification interval is recorded as:

(3) $$\max\frac{2}{\left||w|\right|}$$

The process of finding the optimal classification line is the process of maximizing the classification interval. If the problem is transformed into its dual problem, it can be obtained by using the Lagrange multiplier method. When classifying the test samples with the best classifier from the training set of all flow data sample points, it is very likely that the classifier model over adapts the training sample and classifies all the training data correctly. The fault tolerance of test samples is lower. The support vector machine (SVM) algorithm-based soft margin is used to train the sample set, so that the classifier has better fault tolerance by adding the parameter penalty factor. See Ref. [14] for detailed principles.

To solve the problem of linear separability, we can find the optimal classification line, and map it to high dimensional space to solve the problem of linear inseparability. A support vector machine classifier for nonlinear sample data can be constructed by using kernel function. The kernel function can be calculated directly in the original low-dimensional space without having to represent the mapping of the sample data in the high-dimensional space, which can effectively avoid the complex computation in the high-dimensional space [15,16,17,18]. There are four types of kernel functions commonly used in support vector machines:

Linear kernel function:

$$k(x_{i},x_{j})=x_{i}{}^{T}x_{j}$$

Polynomial kernel function:

$$k(x_{i},x_{j})={\left(x_{i}{}^{T}x_{j}\right)}^{d}\;d>=1$$

Gaussian kernel function:

$$k(x_{i},x_{j})=\exp(-\frac{||x_{i}-x_{j}|{|}^{2}}{2\sigma^{2}})\;$$

Sigmod kernel function:

$$k(x_{i},x_{j})=\tanh(\beta x_{i}{}^{T}x_{j}+\theta )$$

The selection of penalty factor and kernel parameters will affect the performance of the classifier when the SVM algorithm is used to classify the sample data. To make the SVM model better adapt to the training samples and more accurately classify the training samples, the optimal algorithm can be used to optimize the parameters of SVM model. In this paper, an adaptive mutation PSO algorithm is proposed to solve the problem of underfitting and overfitting.

### 2.3. Optimized PSO Algorithm Based on Adaptive Mutation

Particle Swarm Optimization (PSO) is a heuristic algorithm, and also is an optimization algorithm based on swarm intelligence in the field of computational intelligence. The basic concept is derived from the study of artificial life and predatory behavior of birds. PSO algorithm makes each flow sample data determine the fitness value by the objective function, and each data has known the location of the target point and the current position, as well as the best location found by all particles in the whole population. Each particle determines the next step through its own movement experience and the movement experience of other particles. PSO is initialized as a group of random particles and the optimal solution is found by iteration. The velocity and position update iterations are shown in Equations (4) and (5):

(4) $$v_{i}=w*v_{i}+c_{1}*rand()*(pbest_{i}-x_{i})+c_{2}*rand()*(gbest_{i}-x_{i})$$

(5) $$x_{i}=x_{i}+v_{i}$$

where $v_{i}$ is particle velocity, *x_i_* is the current position of the particle, *w* is an inertial factor, *c*_1_, *c*_2_ is a learning factor, *rand* () is a random number between 0 and 1 [19,20,21]. See Ref. [22] for detailed principles.

**Figure 3:**
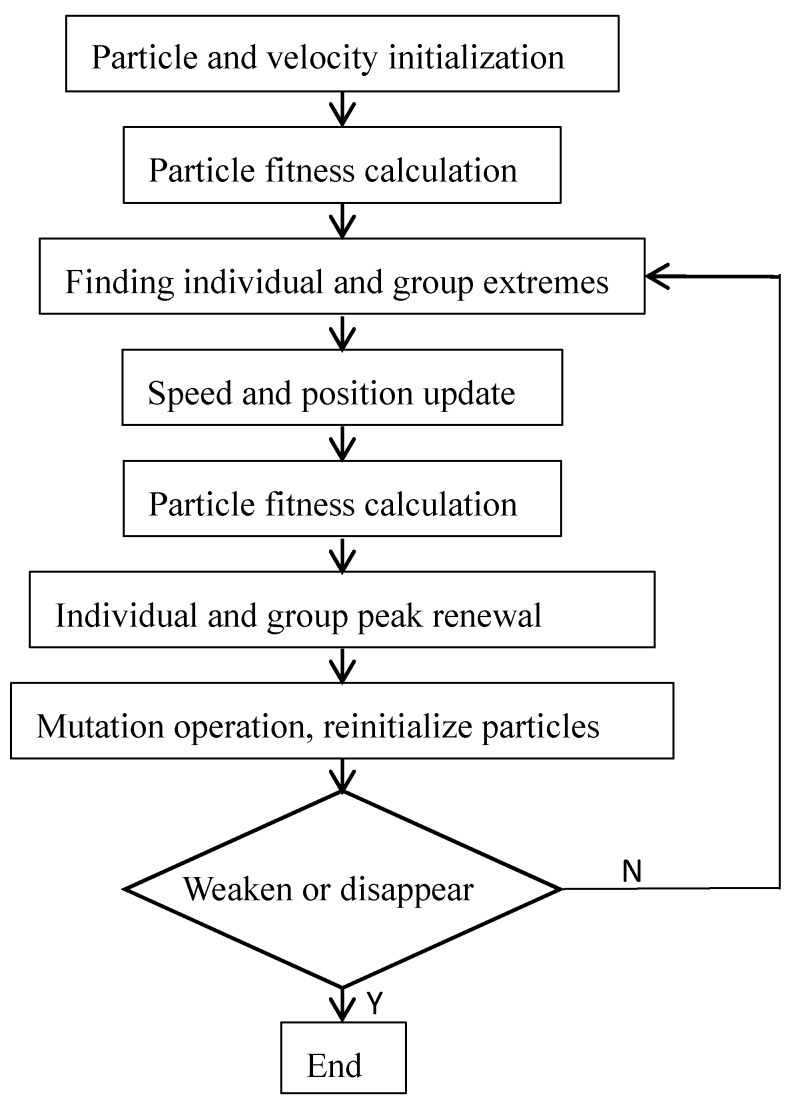
Flow chart of PSO algorithm for adaptive mutation.

The PSO algorithm has the advantages of fast convergence speed and strong generality, but it has the disadvantages of low searching precision, low iterative efficiency and easy to fall into local optimum. Therefore, the mutation operation is introduced into the PSO algorithm, which makes the optimized PSO algorithm reduce the search space of the population continuously in iteration, make it jump out of the local optimum value of the current search, so as to improve the possibility of finding a better value in the algorithm. The algorithm flow is shown in Figure 3. After each iteration optimization, mutation operation is performed and the particle is reinitialized.

**Figure 4:**
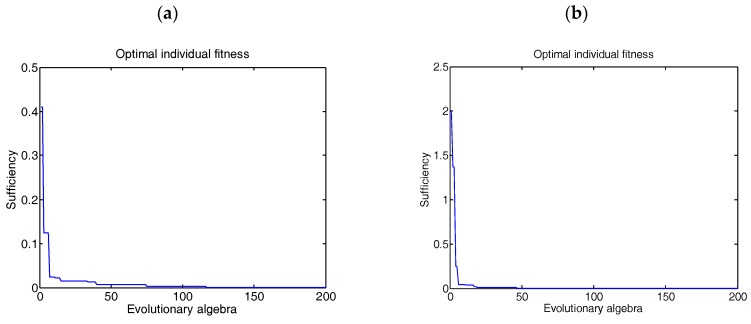
Optimal individual fitness (**a**) PSO algorithm; (**b**) Adaptive mutation PSO algorithm.

To compare the advantages of the adaptive mutation Ackley algorithm with the PSO algorithm, the Ackley function is selected as the fitness evaluation function, which is a continuous experimental function obtained by superimposing the exponent function with the moderately enlarged cosine function. The feature of this function is that the figure is undulating in a curved surface, and the almost flat region is modulated by cosine function to form various holes and peaks [23]. There are many minimum points, the minimum point position is (0, 0), and the fitness function is an Ackley function. The fitness value is the function value. The adaptive mutation PSO optimization algorithm is used to deal with the function, and the optimal individual is obtained. The variation of fitness is shown in Figure 4. It is obvious that the PSO algorithm converges 50 times under the same iteration times, while the adaptive mutation PSO algorithm can jump out of the local optimum and basically converge at about 30 iterations. The speed of convergence has been accelerated.

## 3. SVM Parameter Selection Experiment

**Figure 5:**
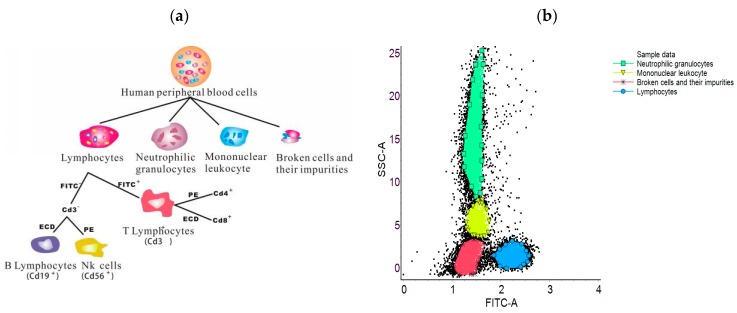
Experimental control group (**a**) Cell staining strategy; (**b**) Artificial clustering results.

Human peripheral blood cells including lymphocytes, neutrophilic granulocytes, Mononuclear leukocyte, broken cells and their impurities, were used in this study. (The experiments is not an interventional clinical study, and it did not need ethic approval information. The aim of this study is to realize the automatic flow cell grouping, which does not involve ethics and morality). The surface molecules were CD3, CD19, CD56 and CD5, respectively, using fluorescein isothiocyanate (FITC), p-phycoerythrin (PE), allophy-cocyanin (APC) and Peridinin-Chlorophyll-Protein Complex (PerCP), respectively. The labeling of orophyll-Protein complex PerCPwas composed of 14 parameters. The lymphocyte group consisted of T lymphocyte B lymphocytes and NK cells, and the cell group staining strategy was as shown in Figure 5a.

The experimental data were measured by FACSCalibur flow cytometry by BD Company (Becton, Dickinson and Company, Franklin, NJ, USA) [24]. The traditional grouping results were obtained by using CytoSpec software of Purdue University (9.0, West Lafayette, IN, USA), and the artificial clustering results were obtained by loop gate. The lateral scattering of light (SSC) reacts to the complexity of the cell, the parameters of FITC-A and SSC of lymphocytes, neutrophils, mononuclear cells, broken cells and their impurities are different. Therefore, these two parameters can be used to distinguish these cells. As shown in Figure 5b, it is used as a standard control for this experiment. The simulation is realized by Matlab R2013b (Natick, MA, USA).

### 3.1. Selection of SVM Kernel Function

Using human peripheral blood cells as experimental data, and a total of 11,324 cell data were obtained. A total of 9 sets of cell data of 400, 800, 1200, …, 3600 were randomly selected for the experiment. Four kernel functions were used: linear kernel function, polynomial kernel function, RBF kernel function and sigmod kernel function, in which the parameters *c* and *g* were chosen as default values. The experimental results of support vector machine training flow data based on different kernel functions are shown in Table 1. The values are respectively the correctness of the algorithm, the number of correct sample test set/the number of samples of test set, and the number of support vectors.

**Figure 6:**
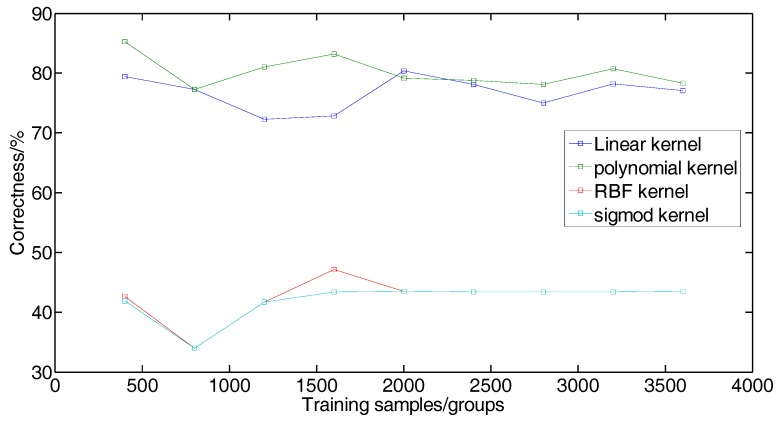
The correctness of four kernel functions.

**Figure 7:**
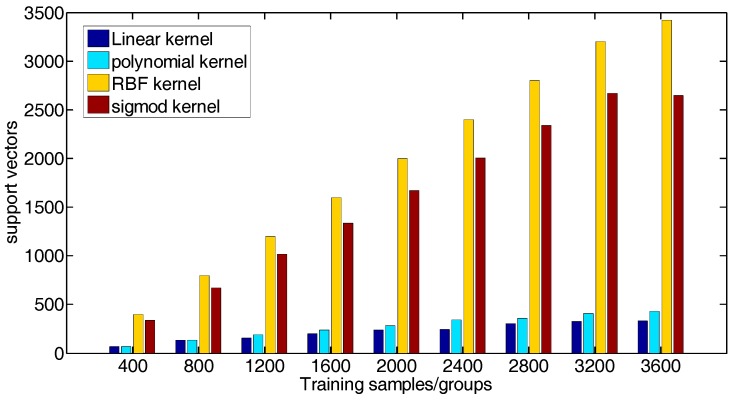
Number of support vectors for four kernel functions.

To compare the advantages and disadvantages of four kernel functions in the processing of flow data more intuitively, the relationship diagram between the number of training samples and the algorithm correctness was drawn according to the results on Table 1, as shown in Figure 6. A histogram is drawn with the number of support vectors obtained from each training model to identify the categories of test samples, as shown in Figure 7.

As can be seen from Figure 6 and Figure 7, the highest correctness rate of processing flow data is obtained by selecting polynomial kernel function, and the number of support vectors in the training model of RBF kernel function is the highest. As shown in Table 2, the correctness of using the default parameter and selecting the polynomial kernel to process the flow data is 80.4044%.

### 3.2. Selection of SVM Model Parameters

Because the correct selection of penalty factors and kernel parameters has a great influence on the results, the default parameters may not be suitable for classification and recognition of flow data. Therefore, the traditional PSO algorithm is used. The parameters of polynomial kernel function and the most commonly used RBF kernel function are selected, and the performance of the two kernel functions trained after parameter optimization is compared. A total of 8 sets of cell data of 400, 800, 1200, …, 3200 were randomly selected for experiments, and the statistical average value was taken as shown in Table 3. It can be seen that the performance of the classifier is greatly improved when the appropriate penalty factors and kernel parameters are selected for the two kernel function SVM models. Compared with RBF kernel function, polynomial kernel function has lower time complexity and less support vector number, so polynomial kernel function is selected to process flow data.

## 4. Experimental Section

### 4.1. Cell Recognition Based on Adaptive Mutation PSO-SVM Algorithm

**Figure 8:**
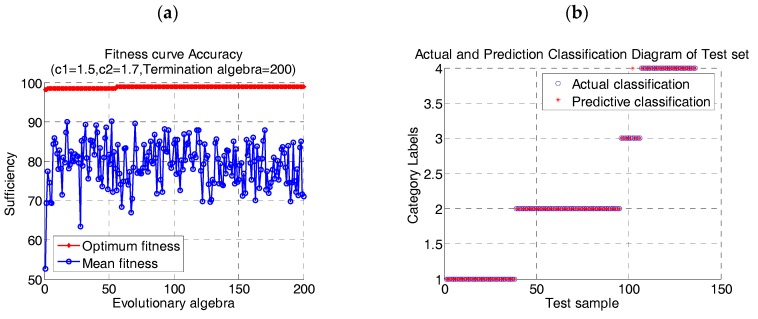
Clustering results of human peripheral blood cells (**a**) Fitness curve; (**b**) Clustering results.

The support vector machine with polynomial kernel function was used as the classifier. The flow data were processed by using the adaptive mutation PSO-SVM algorithm proposed in this paper. The data were selected randomly from 400 groups of human peripheral blood cell samples. Four subsets of cells were extracted according to the proportion of cells in human body. The data of SVM train/test were: lymphocyte 110/36 group, neutrophil 172/57 group, mononuclear leukocyte 30/10 group, Broken cells and impurities 88/30 group. The result of the algorithm is shown in Figure 8.

It can be seen from the diagram that using adaptive mutation PSO algorithm to optimize SVM parameters can effectively avoid population falling into local optimal. When the penalty factor *c* = 4.7682 of SVM model and kernel parameter *g* = 1.4134, the correctness of model training is 98.5%.

**Figure 9:**
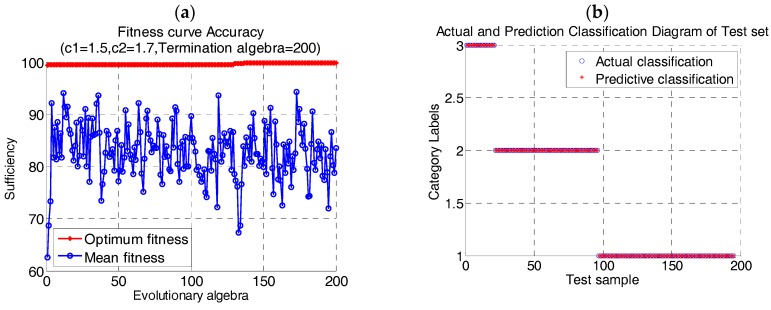
Clustering results of Lymphocyte (**a**) Fitness curve (**b**) Clustering results.

To further verify the effectiveness of the proposed algorithm for flow data clustering, lymphocytes were grouped with the same method and the correctness of clustering was calculated. First, the three groups of lymphocytes were set up by using CytoSpec software, and the label was set as the ideal control group. The three types of lymphocytes contained 3900 groups of data. Support vector machine with polynomial kernel function was selected to classify the data. The parameters of SVM were optimized by adaptive mutation PSO algorithm. 600 groups of data were randomly selected for experiment. The data of train/test were 306/98 groups of T lymphocytes, respectively. The B lymphocyte 232/74 group, the NK cell 62/20 group. The lymphocyte data grouping results was shown in Figure 9. When the penalty factor of SVM model *c* = 15.7647 and the kernel parameter *g* = 3.7827, the correctness of model training was 98.00%.

### 4.2. Discussion

The cross-validation CV algorithm and the adaptive mutation PSO algorithm are used to find the optimal penalty factor *c* and kernel parameter *g* respectively. Then the SVM model is trained by the obtained parameters. In the experiment, a total of 8 sets of cell data of 400, 800, 1200, …, 3200 were randomly selected, and the statistical average values of each index were calculated and compared, as shown in Table 4. Compared with the traditional SVM model, the correctness rate of PSO-SVM algorithm based on adaptive mutation is improved by 19.38%. Compared with the CV algorithm and the PSO-SVM algorithm, the method has higher correctness and lower time complexity.

## 5. Conclusions

The SVM model is established for multi-parameter flow cell group identification. The selection of penalty factor and kernel parameters needs to be set by the operator’s experience, so there are some limitations. In this paper, a PSO algorithm based on adaptive mutation is proposed to solve the parameter optimization problem of SVM model, and the new method is used to deal with multi-parameter flow data. Through the cluster identification of 8 sets of cell data of 400, 800, 1200, …, 3200 human peripheral blood cell sample data, the SVM model is trained with polynomial kernel function, and is optimized by optimization algorithm, compared with the traditional SVM algorithm, the correctness is improved by 19.38%; compared with CV verification and PSO algorithm, adaptive mutation PSO algorithm has higher correctness, and it is 99.79%. Furthermore, the convergence rate can be improved when processing a large number of data. To verify the general applicability of the algorithm, it is used to process lymphocyte data, and the correctness of clustering is 99.12%. The method proposed in this paper is used to process the flow data offline in real time, which promotes the automation of flow cytometry. At the same time, the algorithm have potential application in the biomedical field, such as disease cell analysis and diagnosis, which need to be grouped according to the characteristics.

## Figures and Tables

**Table 1:** Kernel function selection (*c*-1, *g*-0.7).

Group Count	Linear Kernel		Polynomial Kernel		RBF Kernel			Sigmod Kernel	
	Correctness %	SV	Correctness %	SV	Correctness %	SV	Correctness %		SV
400	79.4118 (108/136)	68	85.2941 (116/136)	69	42.6471 (58/136)	400	41.9118 (57/136)		338
800	77.2189 (261/338)	132	77.2189 (261/338)	132	34.0237 (115/338)	800	34.0237 (115/338)		670
1200	75.25 (301/400)	155	81 (324/400)	195	41.75 (167/400)	1200	41.75 (167/400)		1015
1600	72.8464 (389/534)	200	83.1461 (444/534)	237	47.191 (252/534)	1600	43.4457 (232/534)		1336
2000	80.3598 (536/667)	237	79.1604 (528/667)	284	43.4783 (290/667)	2000	43.4783 (290/667)		1670
2400	78.125 (625/800)	245	78.75 (630/800)	342	43.375 (347/800)	2400	43.375 (347/800)		2005
2800	75.0268 (700/933)	302	78.135 (729/933)	359	43.4084 (405/933)	2800	43.4084 (405/933)		2338
3200	78.2364 (834/1066)	328	80.7692 (861/1066)	407	43.4334 (463/1066)	3200	43.4334 (463/1066)		2671
3600	77.0642 (924/1199)	330	78.3153 (939/1199)	428	43.4529 (521/1199)	3420	43.4529 (521/1199)		2649

**Table 2:** Kernel function selection evaluation index.

Kernel	Linear	Polynomial	RBF	Sigmod
Average Correctness %	78.4139	80.4044	42.1814	42.0310
Average SV	212	267	1980	1632

**Table 3:** Comparison of evaluation indexes between polynomial and RBF kernel function.

Order	Kernel	*c*	*g*	CVAcc %	Correctness %	nSVtotal	t/s
1	Polynomial	74.4406	0.1870	99.3219	99.1192	237	497.8755
2	RBF	74.5659	0.1412	99.3174	99.1848	250	589.0584

**Table 4:** Comparison of evaluation index of parameter optimization algorithm.

Order	Algorithm	*c*	*g*	CVAcc %	Correctness %	nSVtotal	t/s
1	SVM	1	0.7	-	80.4044	273	34.6353
2	CV	11.5648	1.3738	99.1797	99.2015	132	505.5738
3	PSO-SVM	82.1829	0.1412	99.3174	99.1848	250	589.0584
4	Adaptive mutation PSO-SVM	74.5659	0.01	99.6629	99.7853	249	489.5275

## References

[1] Ali B., Ryan R.B. (2009). A Survey of Flow Cytometry Data Analysis Methods. Adv. Bioinform..

[2] http://www.kmzehao.com/promotions/recommend/recommend_beckmancoulter_002.html.

[3] Archana S., Elangovan K. (2014). Survey of Classification Techniques in Data Mining. Int. J. Comput. Sci. Mob. Appl..

[4] Li L. (2011). A Survey of Classification algorithms in data Mining. J. Chongqing Norm. Univ..

[5] Deepa S.N., Devi B.A. (2011). A survey on artificial intelligence approaches for medical image classification. Indian J. Sci. Technol..

[6] Xie L.Y., Liu Q., Shao C., Su X.T. (2017). Automatic classification of acute and chronic myeloid leukemic cells with wide-angle label- free static cytometry. Opt. Express.

[7] Kar S., Sharma K.D., Maitra M. (2015). Gene selection from m icroarray gene expression data for classfication of cancer subgroups employing PSO and adaptive K-nearest neighborhood technique. Expert Syst. Appl..

[8] Hasan M.R., Hassand N., Khan R., Kim Y.-T., Iqbal S.M. (2018). Classfication of cancer cells using computational analysis of dynamic morphology. Comput. Methods Programs Biomed..

[9] Tao Y., Wang J., Du J. (2017). Cell recognition based on convolution Neural Network. Chin. J. Med. Phys..

[10] Dosset P., Rassam P., Fernandez L., Espenel C., Rubinstein E., Margeat E., Milhiet P.-E. (2016). Automatic detection of diffusion modes within biological membranes using back- propagation neural network. BMC Bioinform..

[11] Li J., Tseng K.-K., Dong H., Li Y., Zhao M., Ding M. (2014). Human Sperm Health Diagnosis With Principal Component Analysis and K-nearest Neighbor Algorithm. Int. Conf. Med. Biom..

[12] Ghaleb T.A., Mohammed M.A. Automated Analysis of Flow Cytometry Data: A Systematic Review of Recent Methods. Proceedings of the 2016 2nd International Conference on Open Source Software Computing (OSSCOM).

[13] Zhang W.C., Lou X.P., Meng X.C., Zhu L.Q. (2016). Representation Method for Spectrally Overlapping Signals in Flow Cytometry Based on Fluorescence Pulse Time-Delay Estimation. Sensors.

[14] https://en.wikipedia.org/wiki/Supportvectormachine.

[15] Lu S., Jiang M.S., Sui Q.M. (2014). Low Speed Impulse Positioning system of Fiber Bragg grating based on Wavelet transform and support Vector Multiclassifier. Chin. Laser.

[16] Li M., Chen F., Lei M. (2016). Identification of Coal Origin by near Infrared Spectroscopy based on LVQ and SVM algorithm. Spectrosc. Spectr. Anal..

[17] Lu P.F., Fan Y., Zhou L.H. (2017). Extraction and Classification of Animal Blood Spectral Features based on support Vector Machine. Spectrosc. Spectr. Anal..

[18] Bei Y.L., Ren X.N., Peng D. (2013). Particle Swarm Optimization least Squares support Vector Machine combined with partial least Squares method for quality Identification of Sesame Oil. Anal. Chem. Res. Rep..

[19] https://blog.csdn.net/myarrow/article/details/51507671.

[20] Liu Y., Zhang Y., Li D. (2012). Particle Swarm Optimization and Lagrangian Hybrid Optimization methods for extracting Brillouin scattering Spectra from sensors. Chin. Laser.

[21] Zhao Z.G., Zhang J.J., Gou X.F. (2015). Solar Cell temperature Prediction based on Particle Swarm Optimization support Vector Machine. J. Phys..

[22] Tang J. (2010). Principle and Application of PSO algorithm. Comput. Technol. Dev..

[23] https://wenku.baidu.com/view/9e0e6838650e52ea55189870.html.

[24] Ma S.S., Dong M.L., Zhang F. (2017). Study on flow cytometry data clustering based on kernel principal component analysis. J. Biomed. Eng..

